# Peribiliary Cysts Mimicking Primary Sclerosing Cholangitis and Cholangiocarcinoma

**DOI:** 10.7759/cureus.21435

**Published:** 2022-01-19

**Authors:** Shiva Kumar

**Affiliations:** 1 Gastroenterology and Hepatology, Cleveland Clinic Abu Dhabi, Abu Dhabi, ARE

**Keywords:** cholangiocarcinoma, biliary tract, liver transplant, primary sclerosing cholangitis, peribiliary cysts

## Abstract

Peribiliary cysts are cystic dilatations resulting from obstruction of extramural peribiliary glands. They are usually benign, may arise from the intrahepatic and extrahepatic bile ducts, and do not communicate with the biliary tree. They may, however, be confused with alternative diagnoses, resulting in unnecessary investigations with profound clinical implications. We present a case of a 42-year-old male, in whom this entity was misdiagnosed as primary sclerosing cholangitis with cholangiocarcinoma.

## Introduction

Originally described by Nakanuma in 1984, peribiliary cysts are benign cystic dilatations that arise as a result of obstruction of extramural peribiliary glands [1]. These benign lesions may arise from either intrahepatic or extrahepatic bile ducts and typically have no communication with the biliary tree. However, this entity may be confused with alternative diagnoses, resulting in unnecessary investigations, often with significant clinical implications [2,3]. We present the case of a 42-year-old male misdiagnosed with primary sclerosing cholangitis complicated by cholangiocarcinoma, in whom this diagnosis was definitively confirmed by evaluation of explant liver pathology.

This article was previously presented as an abstract at the Annual Scientific Meeting of the American College of Gastroenterology in October 2019.

## Case presentation

A 42-year-old male with a long history of alcohol use was referred for evaluation of possible cholangiocarcinoma complicating underlying primary sclerosing cholangitis with cirrhosis. His clinical presentation was with jaundice, weight loss and imaging evaluation revealed a nodular liver with diffusely beaded appearance of the intrahepatic biliary tree and central hilar prominence suggestive of mass lesion.

**Figure 1:**
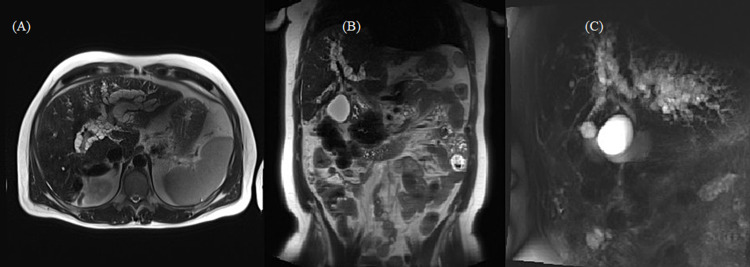
(A and B) MRI with (C) MRCP showed multiple grape-like cystic structures surrounding the intrahepatic portal vein branches MRCP: magnetic resonance cholangiopancreatography

Cholangiographic findings on endoscopic retrograde cholangiopancreatography were suggestive of primary sclerosing cholangitis with suspected cholangiocarcinoma. Cholangioscopy was not performed. However, cytology on biliary brushings showed no evidence of malignancy. Magnetic resonance cholangiopancreatography (MRCP) showed multiple grape-like cystic structures surrounding the intrahepatic portal vein branches, resulting in compression of the intrahepatic bile ducts (Figures 1A-1C).

**Figure 2:**
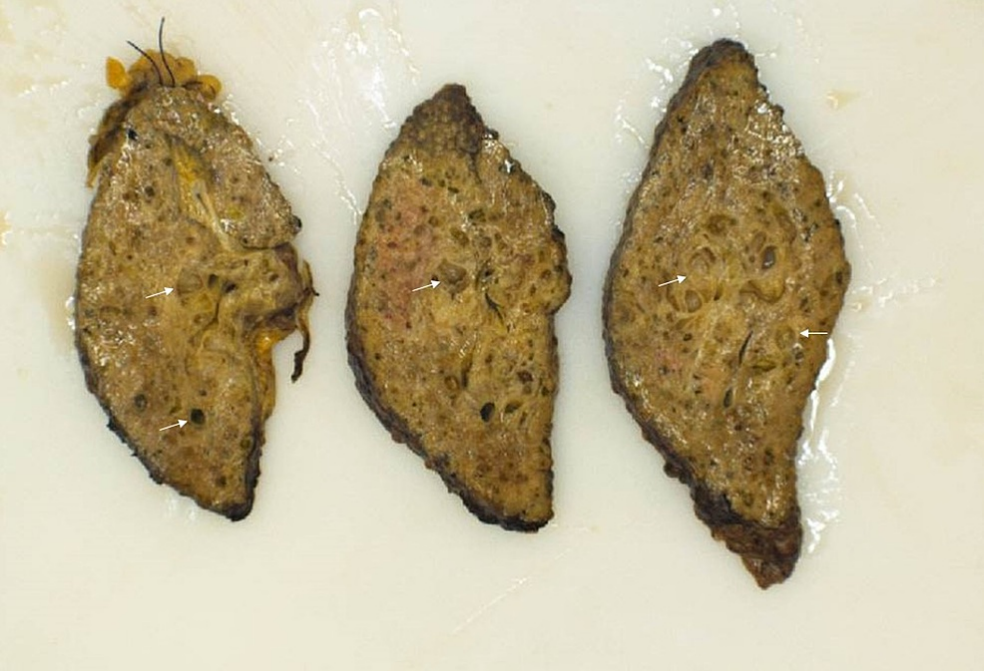
Explant revealed multiple benign bile duct cysts which were focally multicystic (white arrows)

**Figure 3:**
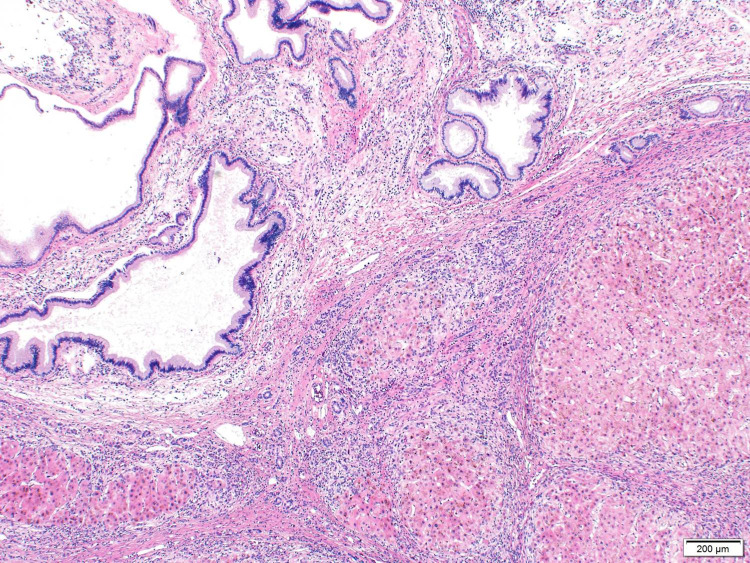
Histology showed multiple cystic biliary structures

**Figure 4:**
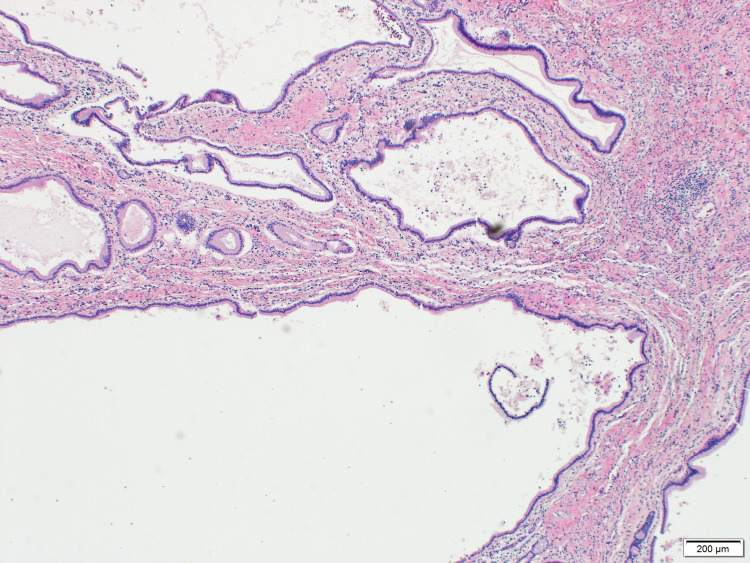
Histologically, cystic biliary structures were lined by non-dysplastic cuboidal biliary type epithelium

These findings were suggestive of peribiliary cysts. Subsequently, due to progressive hepatic decompensation secondary to presumed alcoholic cirrhosis, he successfully underwent deceased donor liver transplantation. Gross examination of the explant revealed multiple benign peri-biliary cysts, which were focally multicystic including the hilum (Figure 2). On histopathology, multiple cystic biliary structures were noted, lined by non-dysplastic cuboidal biliary type epithelium (Figures 3, 4).

## Discussion

Originally described in 1984, peribiliary cysts are usually asymptomatic and often under-diagnosed [1]. Pathogenesis of this entity remains unclear and the cystic dilatations are thought to arise from obstruction of periductal glands external to the biliary walls and therefore do not communicate with the biliary tree. Importantly, this entity may mimic a dilated biliary tree and other clinical entities such as primary sclerosing cholangitis and multi-locular malignancy. Peribiliary cysts could also be mistaken for entities such as biliary intraductal papillary mucinous neoplasm, cystic metastases, Caroli’s disease, and importantly cholangiocarcinoma [2,3]. Peribiliary cysts have been noted to occur at a higher frequency in patients with cirrhosis with a reported prevalence of up to 9% based on imaging criteria [4].

As was the case in our patient, misdiagnosis of peribiliary cysts as cholangiocarcinoma in patients with cirrhosis may result in inappropriate delays and even denial of liver transplant consideration in patients who may otherwise be suitable candidates for liver transplantation. Peribiliary cysts should be considered in patients with cirrhosis who present with intrahepatic biliary dilatations, with or without obstructive jaundice. Pre-transplant, high-resolution imaging with magnetic resonance cholangiopancreatography is often useful in enabling accurate diagnosis, based on the demonstration of cystic structures adjacent to the biliary tree on both sides of the right and left portal veins [2]. Explant pathology often confirms this clinical suspicion. Our case highlights the importance of awareness of this entity in the transplant setting.

## Conclusions

Diagnosis of peribiliary cysts should be considered in patients with cirrhosis who present with dilatation of the biliary tree. Given the prevalence of these lesions in patients with cirrhosis, awareness of this entity is critical to avoid misdiagnosis leading to inappropriate exclusion from transplantation in otherwise acceptable candidates.
